# Alleged Death from Ether

**Published:** 1874-02

**Authors:** Henry J. Bigelow


### SELECTED ARTICLES.

---

### ARTICLE III.

### *Alleged Death from Ether.*

To the Editor of the British Medical Journal:—

Sir: Your issue of October 11th contains the following:—"We have this week to make the sad announcement of a death from the inhalation of ether. It occurred at the South Hants Infirmary. We shall be glad of the comments of Dr. Morgan and our Boston contemporaries."

The avowed interest attaching to death from ether, compared with that attending the rather common occurrence of death from chloroform, attests its rarity; and those who have been long familiar with the safety and efficiency of the former may think it perhaps a little late to subject it in England to an experimental test which the comparative fatality of chloroform seems at length to have secured for it. I venture to comply with the invitation with which you have honored your Boston contemporaries, believing that until some anæsthetic shall be discovered equally safe, with less odor and less bulk, or perhaps some better form of anæsthesia than that by inhalation, ether must be considered on the whole our best anæsthetic. We need not here distinguish too nicely between anæsthesia, narcotism, and inebriation, when effected through the lungs. It is more important that special attention should be directed to several points connected with this subject which seem to be inadequately emphasized in contemporary European literature, especially asphyxia, pulse, and the real difference between what the Journals somewhat promiscuously denominate "death from ether" and "death from chloroform."

The Massachusetts General Hospital numbers more than 15,000 cases of ether inhalation, 6,000 of which have been recorded within the last five years. The quantity of ether consumed during these five years has been about 2,800 pounds,—half a pound, more or less, to a patient; in one case four and a half pounds in twelve hours. It fell to my lot, in 1864, and for a year or two after the discovery of ether anæsthesia, as junior surgeon, to administer most of the ether in that institution; and having been personally cognizant of a large proportion of the cases of its administration there, down to the present time, besides those in my own practice, I have never been satisfied of the occurrence of a single death which could be attributed to any property of ether, apart from the gradual and progressive inebriating influence it possesses in common with other anæsthetic agents.

A detailed report of a case involving so urgent symptoms and so prompt action as the one alluded to, is obviously liable to inaccuracy, and the account should therefore be accepted with reservation, and rather as a good illustration of an emergency quite likely to recur in the experience of those who may believe, with a late English medical journal, that the less the air, during ether inhalation, the better the an-

æsthesia, or, with the French chemist, that nitrous oxide only asphyxiates. Nobody doubts that asphyxia produces insensibility. This is easily shown with a bag containing a few gallons of atmospheric air. But this insensibility, necessarily brief, is unattended by exhilaration. It is distressing, accompanied by lividity, by rigidity if pushed far enough and is doubtless responsible for much of the dread which certain patients have of pulmonary inebriation.

The case in question is reported as follows:—

"David Newman, aged 14, a strumous lad, who had suffered from repeated attacks of corneitis, was admitted an in-patient of the above institution on September 25th, 1873, under the care of Dr. Lake. On Wednesday, October 1st, he was brought into the operating rooms in order that iridectomy might be performed. When on the table, he exhibited considerable alarm, and required some persuasion before he was induced to lie down. Dr. Griffin having taken charge of the pulse, half an ounce of ether was poured on a sponge contained in a cone of spongio-piline, and the latter was closely applied to the mouth and nose. After a few minutes' inhalation, the ether being nearly exhausted, three drachms more were poured on the sponge. Shortly after commencing to inhale this second quantity, he began to struggle violently, getting at length into a state bordering on opisthotonos, his face becoming intensely scarlet. Dr. Griffin then announced that his pulse, which up to this had been perfectly natural, had become very feeble. The ether was at once discontinued, when, the pulse having improved, Dr. Lake operated, no more ether being administered. At the close of the operation, which occupied only a few seconds in its performance, and before the eye could be bandaged, the pulse became imperceptible, the breathing was suspended, and the countenance livid. The tongue was drawn well out of the mouth, and held there; the calves of the legs were vigorously flagellated, and the chest freely slapped with a wet towel. The effect of these measures was to cause the patient to respire freely, to cry out lustily, and to kick about on the table ; but this improvement did not last long,—probably about a minute. The pulse at the wrist did not return, and the breathing again stopped."

Artificial respiration, electricity, etc., were resorted to, but without effect, and the autopsy revealed nothing of importance

In order to be clearly understood, let me here concisely restate this account, as I interpret the phenomena.

A feeble boy was etherized. During this process, though only partially narcotized, he was very completely asphyxiated, and, when nearly dead, was operated on without efforts at resuscitation. When at last his absolute prostration awakened serious alarm, he was vigorously flogged with the view of restoring his exhausted strength ; and under this active stimulus was excited to a final muscular effort which expended and extinguished his flickering vitality. I believe that such a death might have occurred without the ether.

Let us consider the circumstances in detail, and see whether or not they substantiate this hypothesis ; and first, the apparatus employed for inhalation. This was calculated to produce asphyxia.

If the spongio-piline was covered, as usual, with rubber, no air could reach the patient, except in the interstice between the cone and the patient's face. But, according to the account, the cone was closely applied. If so, absolute asphyxia would ensue. It may seem superfluous to say that during the etherizing process a patient must live, as usual, upon oxygen. Let us even needlessly assert that without it a man must die. Ether will not save his life, if he is deprived of oxygen. It would not have saved Desdemona.

Asphyxia did ensue ; but its symptoms passed unheeded. The patient struggled violently. Now a half-conscious struggle often results from mere muscular excitement, and is of little moment. But a rigid struggle, with opisthotonos, is very different. Such spasm is connected with asphyxia, and may involve the muscles of the larynx. In this connection let me remark that there is a wide distinction between the common and desirable snore of a relaxed and vibrating soft palate and a croupy stertor of the contracted laryngeal aperture. The latter is a part of that general rigidity of which opisthotonos is a manifestation, and by excluding air it indefinitely prolongs the asphyxia which occasions it. Air is its only remedy.

These appearances are familiar, in the practice of the Massachusetts General Hospital, even to the house pupils and ward-tenders, to whom etherization is habitually entrusted. Lividity of the forehead indicates a probable similar color of the blood within the head, and announces that spasm may not be far off. Conversely, muscular spasm and laryngeal stertor direct attention to the color of the face. All these symptoms raise the question of admitting air promptly, and until the natural color returns. Indeed, it sometimes happens that because the muscles are rigid a patient seems imperfectly etherized, when the experimental admission of air relaxing the muscles proves the contrary. Even a half-conscious resistance, terminating in insensibility often makes the patient a little livid; so that a struggle then suggests examination of his condition, and sometimes an interval and re-commencement.

If all this be true of inhalation with a sponge, through the meshes of which air has free access to the lungs, and which for hospital use, if not the most economical, is beyond comparison the simplest and safest ether inhaler, what were the chances of a slender boy, struggling desperately for breath, rigidly convulsed with opisthotonos, his face congested, his mouth and nose still sealed by an impervious cone forcibly and closely applied until the pulse gave way?

I unhesitatingly submit asphyxia as the primary cause of death, upon this report.

Notwithstanding this condition of the patient, he was operated on. With so complete asphyxia, it would in Boston be considered of the first importance, before operating, to re-establish respiration, pulse, and color; after which more ether might be administered, to complete the anæsthesia. But in this case no such efforts were made, and no such interval was allowed. After a few seconds which were occupied by the operation of iridectomy, the patient still livid from his struggle with the closely applied cone, "the pulse became imperceptible, the breathing was suspended," and in "about a minute" he was dead.*

To this overwhelming effect of asphyxia upon a slender subject was doubtless added a certain amount of ether inebriation ; but there is abundant evidence that this was but partial and incomplete. The quantity of ether admi istered was inconsiderable; and it is distinctly stated that the patient, when his legs were vigorously flagellated, and the chest freely slapped with a wet towel, "cried out lustily, and kicked about on the table," during the one minute he lived after the operation. Narcotism had not even reached insensibility to pain. No such imperfect ether anæsthesia can be held as principal in such a death.

It would be equally unphilosophical, in view of these facts, and in an endeavor to shift responsibility, to accuse the improbable shock of so slight a surgical operation, and still more any mysterious and as yet undiscovered property of ether, outside of that familiar, gradual and comparatively inocuous influence which it posesses in common with other intoxicating agents. Further reference will be made to this.

A word about restoratives. The most effectual method of resuscitating a patient asphyxiated or over-dosed with ether is at once and quietly to get good air into his lungs. The volatile quality of both chloroform and ether makes their elimination from the pulmonary surfaces so easy, that even when breathing seems to have ceased, a little thoracic movement, artificially assisted, generally enables the patient himself to re-establish respiration, and brings up the pulse.

---

* The fact that "the pulse improved " during a brief interval, does not necesarily modify the general aspect of this case —See *Principles and Practice of Medical Jurisprudence*, by ALFRED SWAINE TAYLOR, M. D., etc., etc. Phila. 1873, Vol. ii. p. 35.

A feeble boy, who had exhausted his strength in a violent struggle for breath and life, would have no great stock in store to respond to a vigorous flagellation. In this respect he might differ from one who had gone tranquilly to sleep with opium.

In arraigning ether, let us not confound things. All powerful therapeutic agents and expedients may, under certain circumstances, contribute to depress the system,—ether and chloroform among the rest; chloroform, as stronger than ether, possessing, of the two, the greater depressing influence. But this effect of a mere narcotism common to both, and which may contribute to the death of a feeble or dying patient, is not the real subject of discussion in the medical journals. The question is, has either of these agents, besides this gradual narcotic power, any additional, different, and peculiar quality, which renders it dangerous? To this I unhesitatingly reply, that chloroform has, and ether has not.

When we say "death from chloroform," we mean death by a shock or poison peculiar to chloroform, even when inhaled by a healthy person, under the most favorable circumstances, with abundance of air, and with every precaution; sometimes occurring at the beginning of anæsthesia undertaken for a trivial operation, almost as if by prussic acid; the sudden failure of a normal pulse indicating that the patient is beyond recovery.

With ether, I believe this to be simply impossible. It always acts slowly, never depressing the vital powers suddenly, or beyond recovery, without fair warning by the pulse in time to avert danger by the simple expedient of filling the lungs with unadulterated air.

In a somewhat extended paper upon anæsthetic agents, written in 1848 at the request of the American Medical Association, and published in the Transactions of that body, about one year and a half after Morton performed his first painless extraction of a tooth, and only a few months after Professor Simpson,s first experiment with chloroform, the absolute necessity of air, the essential indication of the pulse, the difference between the snore of narcotism and the livid stertor of asphyxia, are all specified and insisted on. I may perhaps be pardoned for quoting in conclusion the following passage, which touches the main point of modern ether discussion.

"Ether does not prevent, nor is it to be considered responsible for, the ordinary collapse, resulting, in certain states of the system, after certain injuries and certain operations. The strong argument in behalf of ether is, that so few instances have occurred in which it could be even suspected of agency in fatal results.

"With chloroform the evidence is a little different. Two somewhat remarkable cases of death, occurring during the brief administration of this agent for surgical purposes, at once present themselves,—the Cincinnati case, and that of Mr. Meggison, at Winlaton. In these cases death occurred in about five minutes from the beginning of the inhalation. * * * * These instances suggest a specific cause of danger. This is the sudden impression upon the system of a powerful inebriating agent. Abundant alcoholic stimulus has often produced immediate death; and analogy would suggest that inebriating vapor in the lungs may be the equivalent of similar fluid in the stomach, and that in one or both of the cases alluded to, chloroform may have produced a sudden and overwhelming shock upon the system."*

Your obedient servant,

HENRY J. BIGELOW.

---

### NOTE.

The inodorous and transitory character of anæsthesia by nitrous oxide, notwithstanding its attendant asphyxia, may perhaps recommend it for the brief extraction of a tooth;

---

*Anæsthetic Agents their Mode of Exhibition and Physiological Effects, by Henry J. Bigelow, M. D., one of the Surgeons of the Massachusetts General Hospital.—Transactions of the American Medical Association. Vol. I. 1848.

and we should not ignore the fact that chloroform insensibility is perhaps as safe as many other experiences which people do not hesitate to encounter,—crossing the Atlantic, for example;—and yet one accustomed to the use of ether in surgical operations protracted during an hour or more, with an occasional examination or injury about the pulse, and a suggestion to admit air, if the medical student in attendance happens to forget it, is not a little impressed by the solicitous and apprehensive circumspection attending English anæsthesia.

Under these circumstances, a few practical suggestions, in a familiar form, however superfluous or even trite to a part of the surgical world, may perhaps not inappropriately serve as a record of the current views and practice of etherization in the Hospital with which I am connected,—which has, perhaps, a larger experience than any other, of this form of anæsthesia.

1. Accept the odor and the bulk of ether as a cheap compromise for the safety of the patient and the confidence it gives the operator.

2. Believe that its anæsthetic effects, whether pleasant or objectionable, do not differ materially from those of chloroform.

3. Recognize the fact, while chloroform may kill without warning, ether never does.

4. Aim at anæsthesia by inebriation, not by asphyxia. With ether vapor, insure air to the patient. Though he struggle at the beginning, if he is not rigid or too livid, it is safe to compel inhalation; but if you can devote more time to the process, the resistance will be often less.

(Before etherizing, remove false teeth, and loosen a tight dress.)

5. Use, and let hospital assistants use, a good-sized bell-shaped sponge; and then it may be a question of less rather than more air. The various forms of apparatus which restrict or graduate the quantity of air require more attention and more assistance. Of these a close bag is the worst. If the sponge is damp, it retains ether better, while the vapor is perhaps a little softer than when absolutely pure. The ready ignition of the latter suggests the precaution of moistening with water the skin and saturated linen, before employing near the face even galvano-cautery.

(The gravitation of the vapor makes it practically safe by night, if lamps are held above it.)

6. Keep the pulse in hand; at any rate, examine it often. When the pulse is right, the patient is so. With chloroform, the pulse may be right and the patient wrong. If slow or feeble,* or if the patient snores more than he need, save his strength by giving air,—at any rate, until the pulse comes up; but renew the ether before he is sensible of pain. If the pulse shows that he is suddenly faint, lay him down and give him air. Faintness not unfrequently results from nausea, and is relieved by vomiting. In a case of doubtful pulse, a contractile pupil reassures the operator; a dilated pupil renders him more cautious.

7. If the patient is livid or rigid, give him air.

8. If his glottis contracts, give him air.

10. Should he vomit, of which there is usually timely

---

* "Here is the precaution against danger: . . . . this sign is *the diminution of the force and frequency of the pulse*.

"In an early case of the administration of ether by Dr. Morton, and which has been reported, the danger from over narcotism was quite as imminent as in any case I have since seen alluded to. As a bystander, on that occasion, I casually felt the pulse, and found it barely distinguishable; and though it subsequently still decreased, the means at once adopted for the restoration of the patient proved ultimately successful. This occurrence pointed to the pulse as an index of the stage of narcotism, a few subsequent experiments confirmed the belief; and I have not since hesitated to push etherization to complete insensibility, and to continue it, if necessary, during a length of time, provided the pulse remained full and strong. If it be retarded by ether, it is curious to observe with what certainty it recovers force and frequency, after a few inspirations of pure air. It will be inferred from these remarks that the pulse is to be carefully examined during the whole anæsthetic process, and that inhalation is to be temporarily discontinued at its indication."—*Anæsthetic Agents, etc.*, 1848.

notice, give the matter free exit by turning the patient, if recumbent, well to one side. Although there is less nausea with an empty stomach, it is not well to starve a patient about to encounter a protracted operation.

11. From time to time evacuate the tracheal mucus from the fauces, during an expiration, with a sponge held in dressing forceps.

12. In operations about the nose and mouth, give for convenience, a powerful dose before beginning. Impregnate the whole circulation to the degree it usually attains in the middle of a long operation. The patient is then easily kept quiet. Otherwise a volume of fresh blood may find its way to the brain, and suddenly revive him. Let the repeated dose be also heavy.

13. In these operations, expect blood in the trachea, and evacuate it like the mucus,—but, by reason of its quantity, more promptly.

14. Indeed, if such an operation promises much blood, have a tracheotomy tube ready, with hooks to hold the incision open while they compress the veins, so that the tube can be entered by a cut or two in a few seconds.

15. Or insert the tube before the operation, and put a sponge in the pharynx. The patient may then be etherized through the tube. I have had occasion to resort to these expedients.

16. In artificial respiration, act with the patient, and not against him. He will not cease to breathe at once, and wholly. Enjoin silence; watch the first attempt at inspiration, and at the expiration compress the thorax, aiding its elastic reaction, if absolutely necessary, by Silvester's, or other quiet method. See that the tongue is well forward.

17. Do not cool the patient by exposure and wet surroundings.

18. Being first assured that he can swallow a teaspoonful of water, feed him, if you like, with stimulus, during the expiration, but not the inspiration.

19. Give to all painful surgery, without exception, the benefit of anæsthesia; but a patient unequivocally exhausted by long disease,—of the bladder, or of a joint, for example, —or an habitual inebriate, may require care; without which protracted narcotism may gradually depress his pulse beyond the rallying point. On the other hand, a healthy laborer, who reaches the hospital some hours after a railroad accident, cold, and literally pulseless at the wrist, from hemorrhage and exposure, is, as a rule, stimulated by ether, during and after at least one amputation.

20. Notwithstanding every expedient, there is occasionally an untoward subject who is habitually tetanic and livid, whenever etherized; or, more rarely, one whose respiration is notably intermittent before he becomes insensible. The latter requires attention. In children, it may be added, anæsthesia is cumulative.

Such are some of the minor considerations and prompt precautions which collectively determine the question of life or death in the exceptional emergencies of anæsthesia by ether. Many of them apply with equal force to chloroform; but against the shock of chloroform and its sequences, whether "chloroformic syncope," "cerebral anæmia," or "cerebral congestion," precaution avails nothing.

